# Lysine in Combination With Estradiol Promote Dissemination of Estrogen Receptor Positive Breast Cancer *via* Upregulation of U2AF1 and RPN2 Proteins

**DOI:** 10.3389/fonc.2020.598684

**Published:** 2020-11-30

**Authors:** Gabriela Vazquez Rodriguez, Annelie Abrahamsson, Maria V. Turkina, Charlotta Dabrosin

**Affiliations:** ^1^Department of Oncology, Linköping University, Linköping, Sweden; ^2^Department of Biomedical and Clinical Sciences, Linköping University, Linköping, Sweden

**Keywords:** microdialysis, dissemination, lysine, zebrafish, essential amino acids, breast cancer

## Abstract

The majority of estrogen receptor positive (ER+) breast cancer (BC) maintain the ER at metastatic sites. Despite anti-estrogen therapy, almost 30% of ER+ BC patients relapse. Thus, new therapeutic targets for ER+ BC are needed. Amino acids (AAs) may affect the metastatic capacity by affecting inflammatory cells. Essential AAs (EAAs) cannot be produced by human cells and might therefore be targetable as therapeutics. Here we sampled extracellular EAAs *in vivo* by microdialysis in human BC. Mass spectrometry-based proteomics was used to identify proteins affected after EAA and estradiol (E2) exposure to BC cells. Proteins relevant for patient survival were identified, knocked down in BC cells, and metastatic capability was determined *in vivo* in the transgenic zebrafish model. We found that lysine was the most utilized EAA in human ER+BC *in vivo*. In zebrafish, lysine in presence of E2 increased neutrophil-dependent dissemination of ER+ BC cells *via* upregulation of U2AF1 and RPN2 proteins, which both correlated with poor prognosis of ER+ BC patients in clinical databases. Knockdown of U2AF1 and RPN2 decreased the expression of several cell-adhesion molecules resulting in diminished dissemination. Dietary lysine or its related metabolic pathways may be useful therapeutic targets in ER+ BC.

## Introduction

Amino acids (AAs) participate in all aspects of the pathophysiological processes that occur in tissues and organs (1). One hallmark of cancer is an increased demand for proteinogenic AAs due to extensive proliferation and metabolic reprogramming (2). Moreover, the metastatic capacity of cancer cells might depend on the redirection of AA use (3). The metabolic reprogramming of cancer cells is coupled to inflammation in the tumor microenvironment, leading to increased immune cell infiltration, which further enhances the metastatic capacity of cancer cells (4). Protein biosynthesis requires the local availability of AAs in the extracellular microenvironment, which can also act as signaling molecules that regulate the expression and activity of metastasis-related proteins, such as epithelial-to-mesenchymal transition proteins, matrix metalloproteinases, and focal adhesion kinase phosphorylation (5–8). Thus, AAs in the tissue microenvironment play an important role in controlling the progression of cancer. Essential AAs (EAAs) are AAs that cannot be synthesized by human cells and must be acquired through dietary intake (9). Because of that, EAAs might be useful as potential targets in cancer therapies. Promising results in experimental cancer models have shown that dietary restriction of EAAs can reduce tumor growth and metastasis (8, 10, 11).

Breast cancer (BC) is the most common cancer in women worldwide (12). More than 75% of all BC are estrogen receptor-positive (ER+) (13). Anti-estrogen therapy is the gold standard for patients with ER+ BC; however, approximately 30% of patients that receive anti-estrogen therapy experience relapse, making distant metastasis the leading cause of death among patients with ER+ BC (13). Estrogen stimulates proliferation of ER+ BC leading to an increased demand of AAs. Additionally, estrogen may also affect the influx of innate immune cells into BC tissues and promote a pro-tumoral polarization of these cells (14–16). Patients with various types of cancer, including BC, display altered levels of free proteinogenic AAs in their plasma and saliva (17) but it is not known whether EAA availability is altered in human ER+ BC microenvironment.

We aimed to determine whether EAAs availability is altered in human ER+ BC *in vivo* and, to investigate if the most consumed EAA affected BC cell dissemination. We further aimed to determine what mechanisms are involved in EAA mediated BC cell dissemination.

We found that consumption of five EAAs was increased in human ER+ BC; the levels were lower in BC than in normal breast tissue, with lysine displaying the most pronounced change. In an *in vivo* zebrafish model, exposure to lysine and estradiol (E2) increased the dissemination of human ER+ BC cells. Using mass spectrometry-based proteomic approach, we found that in ER+ BC mammospheres, exposure to lysine and E2 upregulated the splicing factor U2AF 35 kDa subunit (U2AF1) and ribophorin-2 (RPN2) proteins, which both were related to poor prognosis in BC patients. Knockdown of these proteins, achieved by using pre-designed silencing RNAs (siRNA), affected cell proliferation and migration, expression of cell adhesion molecules, and focal adhesion formation resulting in an inhibition of neutrophil-mediated dissemination of ER+ BC cells *in vivo*.

Strategies targeting lysine depletion, uptake, or lysine related pathways might be effective targets in ER+ BC.

## Materials and Methods

### Reagents

DMEM, Opti-MEM, DMEM/F12, DPBS glucose, MEM Vitamins 100 X, MEM Non-essential amino acids (NEAA) 100 X, Glutamine, Penicillin-G/Streptomycin and fetal bovine serum (FBS) were purchased from Gibco™, MA, USA. Bovine serum albumin (BSA) from Merck, NJ, USA. Apo-transferrin, ECM gel, 17-β-Estradiol, Tricaine, hydrocortisone, L-lysine hydrochloride, insulin, urea, iodoacetamide, collagen type I solution and recombinant trypsin from Sigma, MO, USA. Lipofectamine RNAi MAX transfection reagent, heat inactivated FBS and Ethylenediaminetetraacetic acid (EDTA) from Invitrogen, MA, USA. MammoCult kit and heparin from StemCell Technologies Inc., VBC, Canada. Silencer select negative control (#4390843) and silencer pre-designed siRNAs - U2AF1 (siRNA ID#s14553), and - RPN2 (siRNA ID#s230095), restore™ plus Western blot stripping buffer and Fast DiI™ oil red dye from ThermoFisher Scientific, MS, USA. Ficoll-Paque Plus from GE Healthcare, IL, USA.

### Microdialysis of Patients

The regional ethical review board of Linköping approved the study. All participants gave informed consent, and all procedures were performed according to the Declaration of Helsinki. Eleven women (52–78 years of age), who were diagnosed with BC, underwent microdialysis before surgery. Before insertion, 0.5 ml lidocaine (10 mg/ml) was administrated intracutaneously. Microdialysis catheters with 10 mm membrane (#71, M Dialysis AB, Stockholm, Sweden) were inserted intratumorally into the BC and into adjacent normal breast tissue and connected to a pump (M Dialysis AB) and perfused with 154 mM NaCl and 60 g/l hydroxyethyl starch (Voluven; Fresenius Kabi AB, Uppsala, Sweden) at a perfusion rate of 0.5 µl/min. After a 60-min equilibration period, the outgoing perfusate was stored at −80°C.

### Quantifications of Extracellular EAAs in Microdialysis Samples

Ultra-high-performance liquid chromatography with tandem mass spectroscopy and electrospray ionization (UHPLC-ESI-MS/MS) were used for EAAs quantification as previously described (18). Microdialysis samples from all patients were collected at the same time point after the insertion of the microdialysis catheters. 5 µl microdialysis samples were diluted with 20 µl LC-MS grade water (Sigma-Aldrich) and stored at −80°C. When analyzed, samples were thawed at +4°C, then 10 µl of the sample or the EAAs standard were prepared in 20% Voluven and mixed with 8 µl of 0.5 mM perfluoroheptanoic acid (Sigma-Aldrich) in water containing internal standards (Cambridge Isotope Laboratories, Tewksbury, MA, USA). 10 μl of sample was injected and standard curves for each EAA were created and concentrations were calculated.

### Cell Lines

ER+ HER-2 negative BC can be divided into the molecular luminal A and luminal B subgroups where luminal B cancers are associated with increased relapse rates and decreased overall survival (19). To distinguish luminal A from luminal B BC, Ki67 and p53 can be used as determinants (20). Here we used the MCF-7 cells as a model for luminal A BC and the more aggressive p53 mutated T47D cells as a model for ER+ luminal B BC. MCF-7 (ATCC Cat# HTB-22, RRID : CVCL_0031) and T47D (ATCC Cat# HTB-133, RRID : CVCL_0553) were purchased from ATCC. MCF-7 were maintained in DMEM with 10% FBS, 2 mM glutamine and 50 IU/ml/50 µg/ml Penicillin-G/Streptomycin and T47D in Opti-MEM with 4% FBS.

### Isolation of Human Neutrophils

Neutrophils were isolated from venous blood from healthy female donors. Blood samples were diluted 1:3 in PBS with 2 mM EDTA and 0.1% heat-inactivated FBS, mononuclear cells were separated by Ficoll-Paque gradient and discarded, pellets containing neutrophils were diluted in 0.9% NaCl and sedimentated 15 to 20 min with 3% dextran T-500/0.9% NaCl. Residual red blood cells were lysed with hypotonic saline solutions. For *in vivo* analysis, neutrophils were re-suspended in DMEM/F12 complete medium (DMEM/F12 with 0.02% of BSA, 10 µg/ml apo-transferrin and 1 μg/ml insulin) or DPBS glucose medium (DPBS glucose with 1× MEM Vitamins, 1× MEM-NEAA, 0.1% BSA, 2 mM glutamine and Penicillin-G/Streptomycin 50 IU/ml/50 µg/ml) and placed on ice until injections.

### Lysine Treatment in ER+ Breast Cancer Cell Mammospheres

MCF-7 and T47D cells were cultured in low attachment 96-well plates (Sigma) at 37°C in MammoCult medium with heparin 4 µg/ml, hydrocortisone 0.48 µg/ml, Penicillin-G/Streptomycin 50 IU/ml/50 µg/ml and 2.5% ECM gel until mammosphere formation. **l**-lysine 0.146 mg/ml (concentration matched to that found in DMEM medium) ± E2 1 nM treatment was added in DPBS glucose medium free of EAAs to MCF-7 and T47D mammospheres during 5 or 3 days, respectively. Thereafter, mammospheres were lysed with RIPA buffer for proteome analysis. Total protein concentration was measured using Pierce™ BCA protein assay kit (ThermoFisher Scientific).

### Proteome Analysis by Liquid Chromatography-Mass Spectrometry (LC-MS)

Equal amount of protein was loaded onto 10 kDa Microcon centrifugal filters (Merck) and processed according to FASP method as previously reported (21). Trypsin digestion was performed overnight at 37°C at 1:100 to protein ratio. Peptides were desalted by using Pierce C18 tips (ThermoFisher Scientific), resuspended in 0.1% formic acid and quantified by A280 using NanoDrop ND-1000 (Thermo Fisher Scientific, San Jose, CA, USA).

Peptides were separated by reverse phase chromatography at a flow rate of 300 nl/min on EASY-nLC II (Thermo Scientific). Automated online analyses were performed in positive mode by LTQ Orbitrap Velos Pro hybrid mass spectrometer (Thermo Scientific) as described previously (22). Generated files were analyzed using Sequest HT in Proteome Discoverer (Thermo Fisher Scientific, San Jose, USA, v. 1.4.0.288) using Uniprot database for Homo sapiens and searched with a fragment ion mass tolerance of 0.50 Da and parent ion tolerance of 6.0 ppm. Carbamidomethylation of cysteine was specified as fixed modification and oxidation of methionine, acetyl of the N-terminus and phosphorylation of serine, threonine and tyrosine as variable modifications. Scaffold (version 4.9.0; Proteome Software Inc., Portland, OR) was used to validate and quantify identified proteins. Identifications were based on a minimum of 2 peptides, 90% peptide identification probability, and minimum 99% protein identification probability (23). Proteins containing similar peptides were grouped into clusters. The label-free quantitative analysis was performed using total number of spectral counts.

### Bioinformatics Analysis

Significantly altered proteins were analyzed by String database (STRING, RRID : SCR_005223) where Reactome pathways were obtained.

### Transfection Experiments With Silencing RNAs

MCF-7 and T47D were seeded at 5 × 105 cells/well in 6-well plates in DMEM with 10% FBS and 2 mM glutamine. After 24 h incubation, fresh medium with Opti-MEM containing lipofectamine RNAiMAX transfection reagent and 25 pmol of pre-designed silencing RNAs (siRNAs) targeting the genes U2AF1, RPN2, or negative control (siRNA-C) was added. After 72 h incubation at 37°C, cells were used for migration assay, lysed in RIPA buffer for Western blot or labeled with Fast DiI™ oil red dye, 4 µg/ml, for zebrafish experiments as previously described (24).

### Migration and Proliferation Assays

MCF-7 and T47D were transfected with siRNA-C, siRNA-U2AF1 or siRNA-RPN2 respectively, then seeded at 3 × 10**^4^** cells/well in CytoSelect 96-well migration assay plate (Cell Biolabs) in DMEM/F12 complete medium. DMEM with 10% FBS and 2 mM glutamine was used as chemoattractant in the lower chamber. After 24 h incubation at 37°C, cell migration was analyzed using the Spark 10M microplate reader (Tecan Trading AG, Switzerland). For proliferation, 3 x 10^3^ cells were seeded/well in 96-well plates and transfected as above. BrdU cell proliferation assay (Roche Cat#11 647 229 001) was used and proliferation was measured with VersaMax microplate reader (Molecular Devices, California, USA).

### Western Blot

Cell lysates were loaded onto 4% to 15% SDS-PAGE gels (BioRad), and separated proteins were transferred to PVDF membranes (BioRad), which were incubated with primary antibodies; mouse anti-human VCAM-1 1:1000 (Novus Cat# NBP1-28404, RRID : AB_1852860), mouse anti-human ICAM-1 1 µg/ml (Novus Cat# NBP2-22541), mouse anti-human MUC-1 1 µg/ml (Novus Biologicals Cat# NBP2-34737), mouse anti-human RPN2 1:300 (Santa Cruz Biotechnology Cat# sc-166421, RRID : AB_2238716) and mouse anti-human U2AF1 1:200 (Nordic BioSite Cat# LS-C751868). Goat anti-rabbit HRP 1:2000 (Agilent Cat# P0448, RRID : AB_2617138) and goat anti-mouse HRP 1:2500 (Agilent Cat# P0447, RRID : AB_2617137) were used as secondary antibodies. Rabbit anti-human GAPDH 1:2500 (Abcam Cat# ab9485, RRID : AB_307275) was used as loading control. Proteins were detected with ECL prime Western blotting detection reagent (GE Healthcare), visualized by Chemidoc™ MP Imaging System and analyzed with Image Lab™ Software version 5.2.1 (Image Lab Software, RRID : SCR_014210). Western blot images in the main manuscript were created by cropping and pasting from the original images shown in full in **Supplementary Figure 1**.

### Immunocytochemistry

Cells were cultured at 2 × 10**^5^** cells/well on collagen-coated cover glasses in 6-well plates, transfected with siRNAs as described above and fixed in 4% PFA. Mouse anti-human vinculin 2 µg/ml (Thermo Fisher Scientific Cat# MA5-11690, RRID : AB_10976821) was used as primary antibody and goat anti-mouse Alexa Fluor-488 1:500 (Abcam Cat# ab150113, RRID : AB_2576208) as secondary antibody. Glasses were mounted with SlowFade Gold antifade reagent with DAPI (Invitrogen) and visualized by confocal microscopy using a Zeiss LSM700 upright confocal microscope (Carl Zeiss AG, Oberkochen, Germany). Focal adhesions area at the invasive front was quantified by using Fiji software version 1.52n (Fiji, RRID : SCR_002285) (25).

### Zebrafish Tumor Xenograft Model

Zebrafish experiments were approved by the animal ethics committee at Linköping University. Transgenic Tg(fli1:EGFP)^y1^ zebrafish embryos, were kept at 28°C in E3 embryo medium with 0.2 mM 1-phenyl-2-thiourea (PTU).

For lysine experiments, MCF-7 and T47D were treated ± E2 1 nM for 48 h, Dil-labeled and treated ± lysine 0.146 mg/ml ± E2 1 nM in DPBS glucose medium for 5 h. Cells were injected in two days old embryos ± lysine 0.146 mg/ml ± E2 1 nM ± 50% neutrophils.

For experiments with transfected cells, MCF-7 and T47D were transfected with siRNAs and cultured in complete media containing lysine in presence of E2 1 nM and Dil-labeled. Cells were injected in two days old embryos + E2 1 nM ± 50% neutrophils.

T47D were treated for 48 h with E2 1 nM, Dil-labeled and injected + E2 1 nM ± isotype antibody control 1 µg/ml (BioLegend Cat# 400166, RRID : AB_11146992) ± mouse anti-human MUC-1 antibody 1 µg/ml as previously described (24).

After 24 h incubation at 28°C in E3/PTU medium ± E2 1 nM, cell dissemination to the tail region was quantified and embryos were photographed in Olympus BX43 light/fluorescence microscope (10×/0.30 magnification) with excitation filters BP460–495 and BP530–550, using Olympus DP72 CCD camera. Images were acquired with the Olympus CellSens Imaging software version 1.16 (Olympus cellSens Software, RRID : SCR_016238).

### Breast Cancer Database Analysis

Kaplan Meier-plotter (KMP) database (Kaplan Meier Plotter, RRID : SCR_018753) (26) was used to analyze overall survival of ER+ BC patients. Patient’s data was split by Q1 vs Q4, overall survival, 180 months of follow up threshold and ER+ BC luminal A and luminal B options were selected, ER+ status derived from gene expression data was included. All other options were left as default.

### Statistical Analysis

Data are presented as mean ± SEM. For multiple group testing ANOVA was used and if significant followed by two-tailed Student t test otherwise two-tailed Student t test was used. The human data was analyzed with Wilcoxon signed-rank test for paired observations. A P value < 0.05 was considered statistically significant. Statistics were performed with Prism 7.0 (GraphPad Software, San Diego, CA).

Analysis of proteomic data was performed with two-tailed Student’s t-test and stack of P values were analyzed with Benjamini-Hochberg correction to assess false discovery rate (FDR), which was set to 5%.

## Results

### Significantly Increased Consumption of Lysine in ER+ BC in Patients

**Figure 1:**
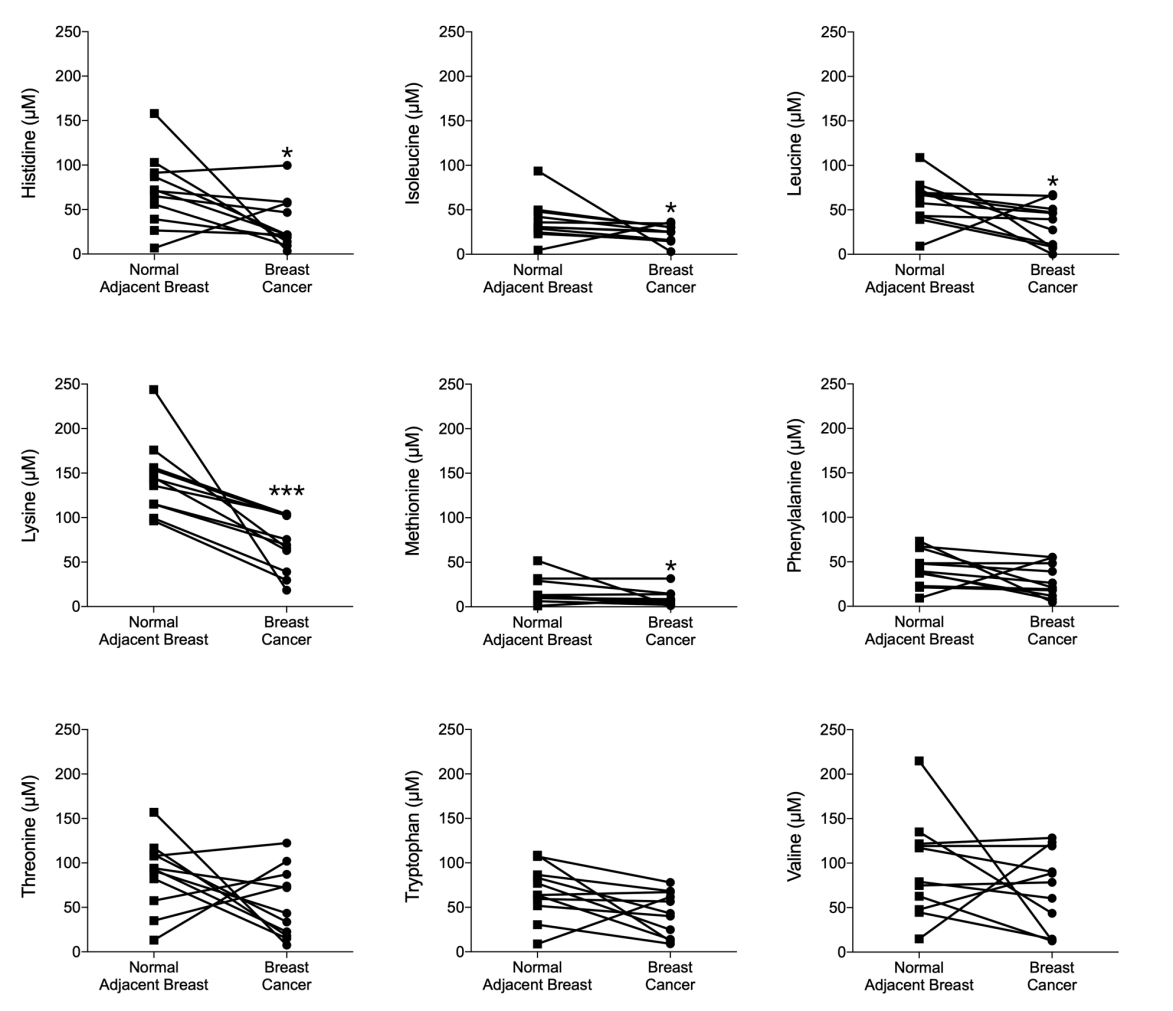
Lysine was the most consumed essential amino acid (EAA) *in vivo* in ER+ breast cancer patients. Microdialysis was used for sampling of extracellular essential AAs (EAAs) from the live tissue of ER+ BC patients before surgery. One catheter was inserted within the breast cancer and another in normal adjacent breast tissue. EAAs were quantified by UHPLC-ESI-MS/MS as reported in materials and methods. Wilcoxon signed-rank test, *P < 0.05 and ***P < 0.001.

Extracellular EAAs from live ER+ BC were sampled using microdialysis in women before surgery. Cancer characteristics are shown in **Table 1**. As shown in **Figure 1**, histidine, isoleucine, leucine, methionine and lysine exhibited significant decreased levels in the cancerous breast tissue compared to normal adjacent breast tissue. Lysine was the most abundant EAA in the extracellular space. Additionally, all BC exhibited decreased levels of lysine whereas the results of the other EAA were more mixed; increased, decreased as well as no change of the levels were detected. Because of this, the effects of lysine were further investigated experimentally.

**Table 1:** Characteristics of the breast cancers of patients subjected to intratumoral microdialysis.

Patient	Age	Tumor size	Grade (NHG)	ER (%)	PR (%)
1	69	21	2	>50	10–50
2	70	22	2	>50	>50
3	68	24	2	>50	>50
4	52	25	3	>50	10–50
5	78	28	2	>50	>50
6	62	21	2	>50	>50
7	48	30	2	>50	>50
8	73	30	2	>50	<5
9	57	27	1	>50	>50
10	66	60	2	>50	>50
11	65	50	2	>50	0

All cancers were HER-2 negative.

NHG, Nottingham grade; ER, estrogen receptor; PR, progesterone receptor.

### Lysine Increased the Neutrophil-Dependent BC Cell Dissemination in Presence of E2 in Luminal A and Luminal B Breast Cancer

**Figure 2:**
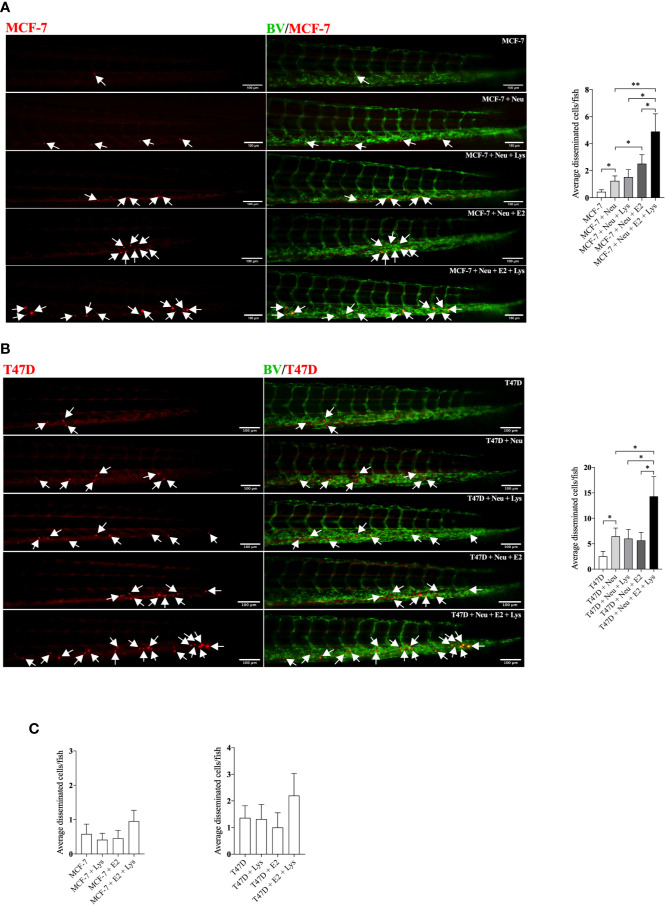
Lysine in combination with estradiol increased breast cancer cell dissemination in presence of neutrophils *in vivo*. ER+ BC cells were pre-treated ± estradiol (E2) ± lysine (Lys). Zebrafish transgenic embryos, with green fluorescent blood vessels, were injected with BC cells ± Lys ± E2 ± neutrophils (Neu) and analyzed as described in materials and methods. **(A)** *In vivo* dissemination of co-injected MCF-7 ± Neu in presence of Lys, E2 or their combination E2+Lys. One-way ANOVA (*P <* 0.0001) followed by two-tailed Student’s t-test, (n = 15–33), *P < 0.05 and **P < 0.01. **(B)** *In vivo* dissemination of co-injected T47D ± Neu in presence of Lys, E2 or their combination E2+Lys. One-way ANOVA (*p* = 0.0014) followed by two-tailed Student’s t-test, (n = 15–22) *P < 0.05. **(C)** *In vivo* dissemination of MCF-7 and T47D cells alone in presence of Lys, E2 or their combination E2+Lys. No significant changes were detected. Representative images of zebrafish embryos are shown, arrows show disseminated BC cells. BV = blood vessels. Scale bar = 100 µm. Data are presented as Mean ± SEM. Data are represented of at least two independent experiments.

As neutrophils are the first responders in the inflammatory process and have been reported as key mediators of BC metastasis (27, 28), we injected BC cells in presence of neutrophils in zebrafish and exposed the embryos to E2, lysine or their combination. As shown in **Figures 2A, B**, both the low-metastatic ER+ luminal A MCF-7 cells and the more aggressive ER+ T47D luminal B cells showed significantly increased dissemination with lysine exposure in presence of E2, compared to untreated controls, lysine or E2 alone.

### Lysine in Presence of E2-Induced Expression of Splicing Factor U2AF 35 kDa Subunit in Luminal A BC Cells

**Figure 3:**
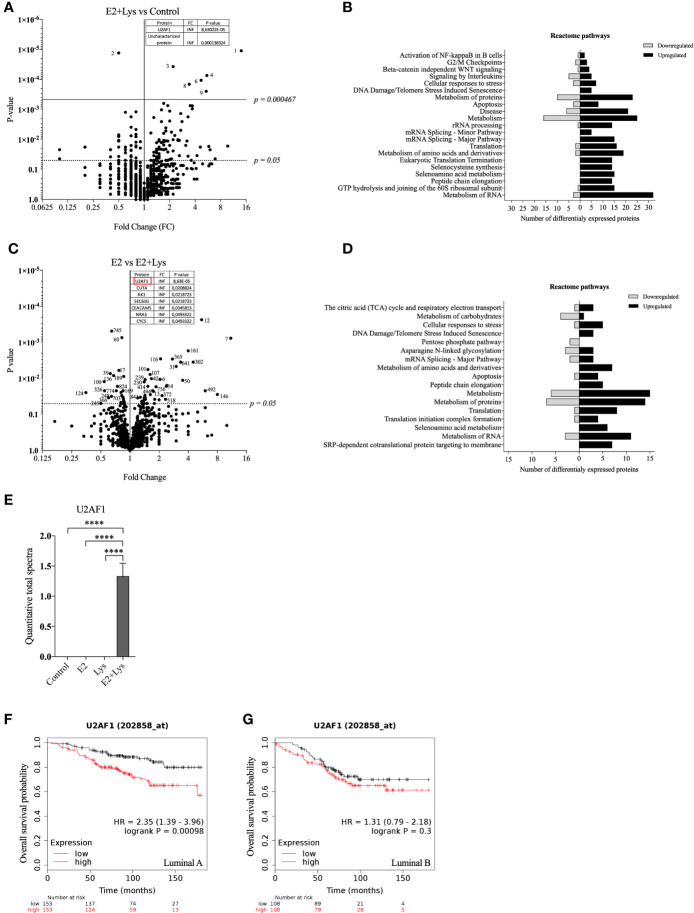
Lysine in presence of estradiol induced expression of U2AF1 protein in luminal A ER+ breast cancer cells. Proteome analysis was performed in non-metastatic luminal A ER+ MCF-7 mammospheres, treated ± lysine (Lys) ± estradiol (E2), using LC-MS as described in materials and methods. **(A)** Volcano plot of identified proteins in E2+Lys vs control treated mammospheres (n = 6). Proteins with infinite (INF) fold change are shown in a separate table. Two-tailed Student’s t-test (*P <* 0.05) followed by False discovery rate method of Benjamini and Hochberg (*P* = 0.000467). **(B)** Reactome analysis of significantly down- and upregulated proteins (*P <* 0.05) by E2+Lys treatment compared to control in mammospheres. **(C)** Volcano plot of identified proteins in E2 vs. E2+Lys treated mammospheres (n = 6). Proteins with infinite (INF) fold change are shown in a separate table, U2AF1 protein is highlighted in a red rectangle. Two-tailed Student’s t-test analysis (*P <* 0.05). **(D)** Reactome analysis of significantly down- and upregulated proteins (*P <* 0.05) by E2+Lys treatment compared to E2 in mammospheres. **(E)** Quantitative total spectra of U2AF1 protein (n = 6). Data are presented as mean ± SEM. Two-tailed Student’s t-test ****P < 0.0001. The Kaplan-Meier plotter (KMP) database was used to analyze the effect of U2AF1 expression in overall survival of ER+ BC patients as described in materials and methods. **(F)** mRNA expression of U2AF1 in ER+ luminal A (n = 306) and **(G)** ER+ luminal B BC (n = 216). HR = hazard ratio. Volcano plots show code numbers corresponding to differentially affected proteins, protein names with their respective code number are shown in **Supplementary Data 1**.

To further elucidate by which mechanisms lysine in combination with E2 increased cancer cell dissemination we next compared the proteome of ER+ BC mammospheres cultured in presence or absence of E2, lysine, and their combination by using LC-MS. As shown in **Figure 3A** and **Supplementary Data 1** a total of 973 single proteins and protein clusters were identified in the luminal A BC cell line MCF-7.

As the most significant changes in cancer cell dissemination occurred when lysine was added in presence of E2 (E2+lysine) compared to untreated control cells we first compared these two groups. Exposure of E2+lysine compared to untreated control mammospheres resulted in significantly changed levels (*P <*0.05 before FDR correction) of 135 single proteins or protein clusters (**Figure 3A**). The Reactome analysis revealed that E2+lysine treatment increased the expression of proteins involved in metabolism including biological pathways related to translation, mRNA splicing and inflammation compared to control treatment (**Figure 3B**).

To clarify the role of lysine, E2+lysine treated mammospheres were compared to mammospheres treated with E2 only. As shown in **Figure 3C** and **Supplementary Data 1**, a total of 80 proteins or protein clusters were significantly changed (*P <*0.05 before FDR correction). Reactome analysis revealed that E2+lysine, increased the expression of proteins involved in mRNA splicing, the TCA cycle and most of BC cell metabolic pathways such as protein, AAs, and RNA metabolism whereas metabolism of carbohydrates was decreased (**Figure 3D**).

FDR correction of the first set of data revealed 9 significantly altered proteins or clusters of proteins in the E2+lysine treated mammospheres compared to controls without treatment (**Figure 3A**). Out of these, 3 remained significantly upregulated in E2+lysine treated mammospheres when they were compared to mammospheres treated with E2 alone; the splicing factor U2AF 35 kDa subunit (U2AF1), the cluster of neuroblast differentiation-associated protein AHNAK, and one uncharacterized protein (**Figure 3C**) suggesting that the changes of these proteins were dependent on lysine.

**Figure 3E** shows that U2AF1 was expressed in the presence of E2+lysine and not in any other treatment groups or control group. To elucidate whether U2AF1 affected BC prognosis in patients the publicly available database “Kaplan-Meier plotter” (KMP) was used. Increased expression of U2AF1 was associated with worse overall survival in luminal A BC but not luminal B BC (**Figures 3F, G**). As clusters of proteins have inherently lower probability of defining a specific protein, U2AF1, as the only identified specific protein, was selected for further experimental studies of luminal A BC dissemination.

Volcano plots and Reactome analysis of lysine vs. control and E2 vs. control groups are provided in **Supplementary Figures 2A to D**.

### U2AF1-Mediated Migration and Dissemination of Luminal A ER+ Breast Cancer Cells

**Figure 4:**
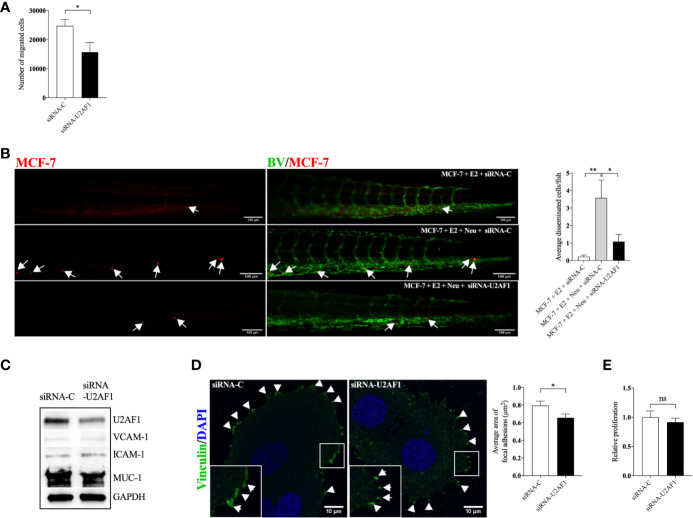
Knockdown of U2AF1 decreased luminal A BC cell dissemination *in vivo*. Luminal A ER+ MCF-7 cells, transfected with negative control siRNA (siRNA-C) or siRNA targeting U2AF1 (siRNA-U2AF1) were injected in presence of estradiol (E2) ± neutrophils (Neu) into zebrafish transgenic embryos, with green fluorescent blood vessels, and analyzed as described in materials and methods. **(A)** Migration *in vitro* (n = 6–12). **(B)** *In vivo* dissemination in presence of E2 ± Neu (n = 38–41). Scale bar = 100 µm. **(C)** Western blot analysis for confirmation of siRNA-U2AF1 transfection and ICAM-1, VCAM-1, and MUC-1 expression. **(D)** Focal adhesion area (n = 7). Scale bar = 10 µm. **(E)** Proliferation *in vitro* (n = 12). Representative images of zebrafish embryos with disseminated MCF-7 cells and immunocytochemistry analysis of vinculin expression are shown. Arrows show disseminated MCF-7 cells and arrowheads show focal adhesions. BV = blood vessels. Data are presented as mean ± SEM. Two-tailed Student’s t-test *P < 0.05, **P < 0.01, ns, not significant. Data are represented of at least two independent experiments.

Next, we set up experiments to evaluate if, and by which mechanisms, U2AF1 protein affected growth and dissemination capacities of ER+ luminal A BC cells, MCF-7 cells. As shown in **Figures 4A, B**, knockdown of U2AF1 significantly decreased MCF-7 cell migration *in vitro* and *in vivo*. In zebrafish, a significant decrease in neutrophil-induced dissemination was observed. As cancer cell and neutrophil interactions are mediated by the expression of cell adhesion proteins, several of these were analyzed in transfected cells. As shown in **Figure 4C**, knockdown of U2AF1 decreased mucin-1 (MUC-1) expression whereas vascular cell adhesion molecule-1 (VCAM-1) or intercellular adhesion molecule-1 (ICAM-1) were unaffected. Overexpression of MUC-1 in MCF-7 cells has previously been shown to be important for invasiveness and metastatic behavior of these cells (29, 30).

Focal adhesions formation has been shown to mediate cell migration, which is key for cancer metastasis (31). **Figure 4D** shows that siRNA-U2AF1 transfection significantly decreased the focal adhesions area at the migratory front of MCF-7 cells. siRNA mediated knockdown of U2AF1 had no effect in MCF-7 proliferation (**Figure 4E**).

### Lysine in Presence of E2 Exposure Increased Ribophorin-2 Protein in Luminal B BC Cells

**Figure 5:**
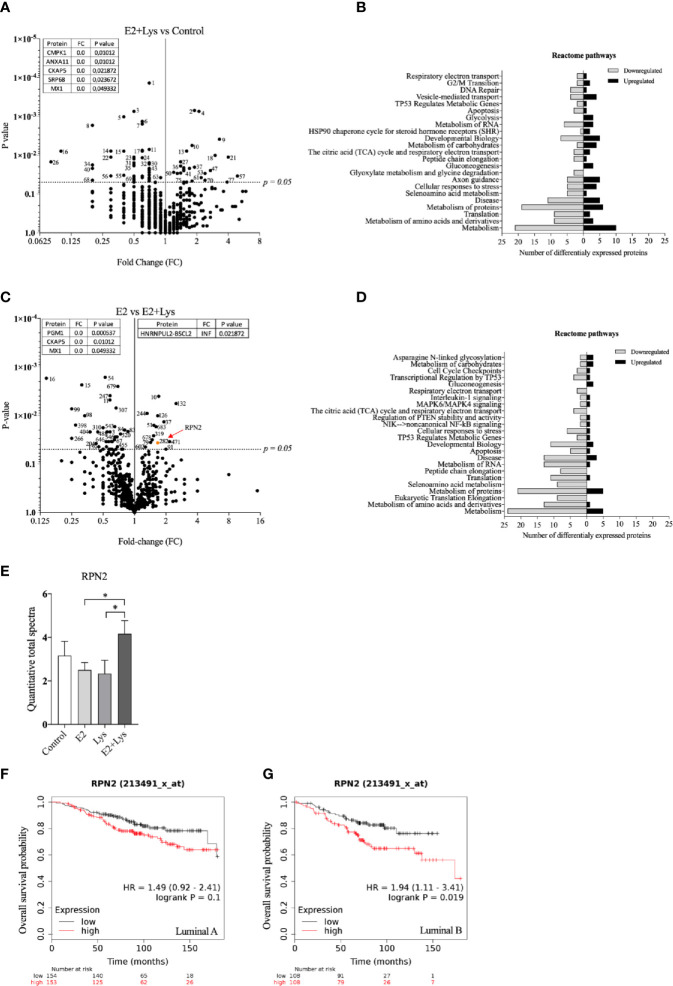
Lysine in presence of estradiol increased RPN2 protein in luminal B ER+ breast cancer cells. Proteome analysis was performed in mammospheres with luminal B ER+ T47D cells, treated ± lysine (Lys) ± estradiol (E2), using LC-MS as described in materials and methods. **(A)** Volcano plot of identified proteins in E2+Lys vs control treated mammospheres (n = 6). Proteins with zero-fold change are shown in a separate table. Two-tailed Student’s t-test (*P <* 0.05). **(B)** Reactome analysis of significantly down- and upregulated proteins (*P <* 0.05) by E2+Lys treatment compared to control mammospheres. **(C)** Volcano plot of identified proteins in E2 vs. E2+Lys treated mammospheres (n = 6). Proteins with zero and infinite (INF) fold change are shown in separate tables. RPN2 protein is highlighted in orange and red arrow. Two-tailed Student’s t-test analysis (*P <* 0.05). **(D)** Reactome analysis of significantly down- and upregulated proteins (*P <* 0.05) by E2+Lys treatment compared to E2. **(E)** Quantitative total spectra of RPN2 protein (n = 6). Data are presented as mean ± SEM. Two-tailed Student’s t-test *P < 0.05. The Kaplan-Meier plotter (KMP) database was used to analyze the effect of RPN2 expression in overall survival of ER+ BC patients as described in materials and methods. **(F)** mRNA expression of RPN2 in ER+ luminal A (n = 307) and **(G)** ER+ luminal B BC (n = 216). HR = hazard ratio. Volcano plots show code numbers corresponding to differentially affected proteins, protein names with their respective code number are shown in **Supplementary Data 2**.

In the luminal B ER+ T47D mammospheres, a total of 765 proteins or protein clusters were identified (**Supplementary Data 2**) and out of these, 83 were significantly altered between the E2+lysine group compared to control mammospheres without treatment (**Figure 5A**). The Reactome analysis revealed that E2+lysine treatment decreased the expression of proteins related to most metabolic pathways whereas it increased the expression of proteins that participate in carbohydrate metabolism and gluconeogenesis (**Figure 5B**). To elucidate which proteins that were affected by lysine, we compared E2+lysine treated mammospheres vs. E2 alone and found 65 proteins or protein clusters that were significantly altered (**Figure 5C**). The Reactome analysis showed decreased expression of proteins related to metabolic pathways while the gluconeogenesis pathway was upregulated (**Figure 5D**). Surprisingly, after FDR analysis no proteins remained significant. Therefore, out of the 65 significantly changed proteins, we identified proteins having an expression level ±50% change in the E2+lysine group compared to E2 alone. Thereafter we search in the KMP database if any of these proteins correlated with poor overall survival in patients with luminal B BC but not with luminal A BC. By this approach the ribophorin-2 (RPN2) protein was identified. As shown in **Figure 5E**, E2+lysine exposure increased RPN2 levels compared to E2 and lysine alone. In **Figures 5F, G**, the role of RPN2 in survival of luminal B BC patients but not for luminal A BC is shown.

Volcano plots and Reactome analysis of lysine vs. control and E2 vs. control are provided in the **Supplementary Figures 2E to H**.

### RPN2-Mediated Proliferation and Dissemination of Luminal B ER+ Breast Cancer Cells

**Figure 6:**
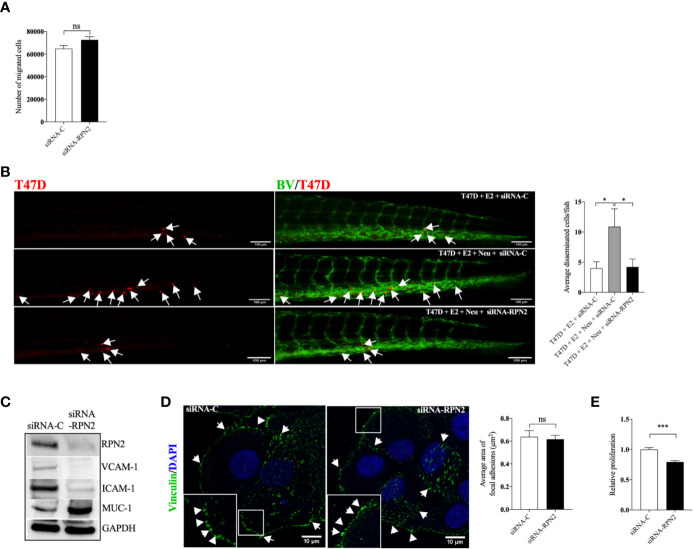
Knockdown of RPN2 decreased luminal B BC cell dissemination *in vivo*. Luminal B ER+ T47D cells, transfected with negative control siRNA (siRNA-C) or siRNA targeting RPN2 (siRNA-RPN2) were injected + estradiol (E2) ± neutrophils (Neu) into zebrafish transgenic embryos with green fluorescent blood vessels and analyzed as described in materials and methods. **(A)** Migration *in vitro* (n = 6). **(B)** *In vivo* dissemination of transfected T47D in presence of E2 ± Neu (n = 23–26). Scale bar = 100 µm. (n = 23–26). **(C)** Western blot analysis for confirmation of siRNA-RPN2 transfection and VCAM-1, ICAM-1, and MUC-1 expression. **(D)** Focal adhesions area (n = 5–6). Scale bar = 10 µm. **(E)** Proliferation (n = 6). Representative images of zebrafish embryos with disseminated luminal B T47D BC cells and immunocytochemistry analysis of vinculin expression are shown. Arrows show disseminated T47D and arrowheads show focal adhesions. BV = blood vessels. Data are presented as mean ± SEM. Two-tailed Student’s t-test *P < 0.05, ***P < 0.001, ns, not significant. Data are represented of at least two independent experiments.

In the more aggressive ER+ T47D, siRNA mediated knockdown of RPN2 had no effect in T47D migration *in vitro*, however, it significantly inhibited the neutrophil-induced dissemination of T47D cells *in vivo* in zebrafish (**Figures 6A, B**). **Figure 6C** shows that knockdown of RPN2 reduced the expression of ICAM-1 and VCAM-1 integrins whereas it increased the expression of MUC-1 in T47D. However, MUC-1 seems to be less important for dissemination of these cells as antibody neutralization of MUC-1 in T47D cells did not affect the dissemination *in vivo* in zebrafish (**Supplementary Figure 3**). **Figure 6D** shows that focal adhesions area was not affected when RPN2 was knocked down in T47D. siRNA mediated knockdown of RPN2 decreased significantly T47D proliferation *in vitro* (**Figure 6E**).

## Discussion

**Figure 7:**
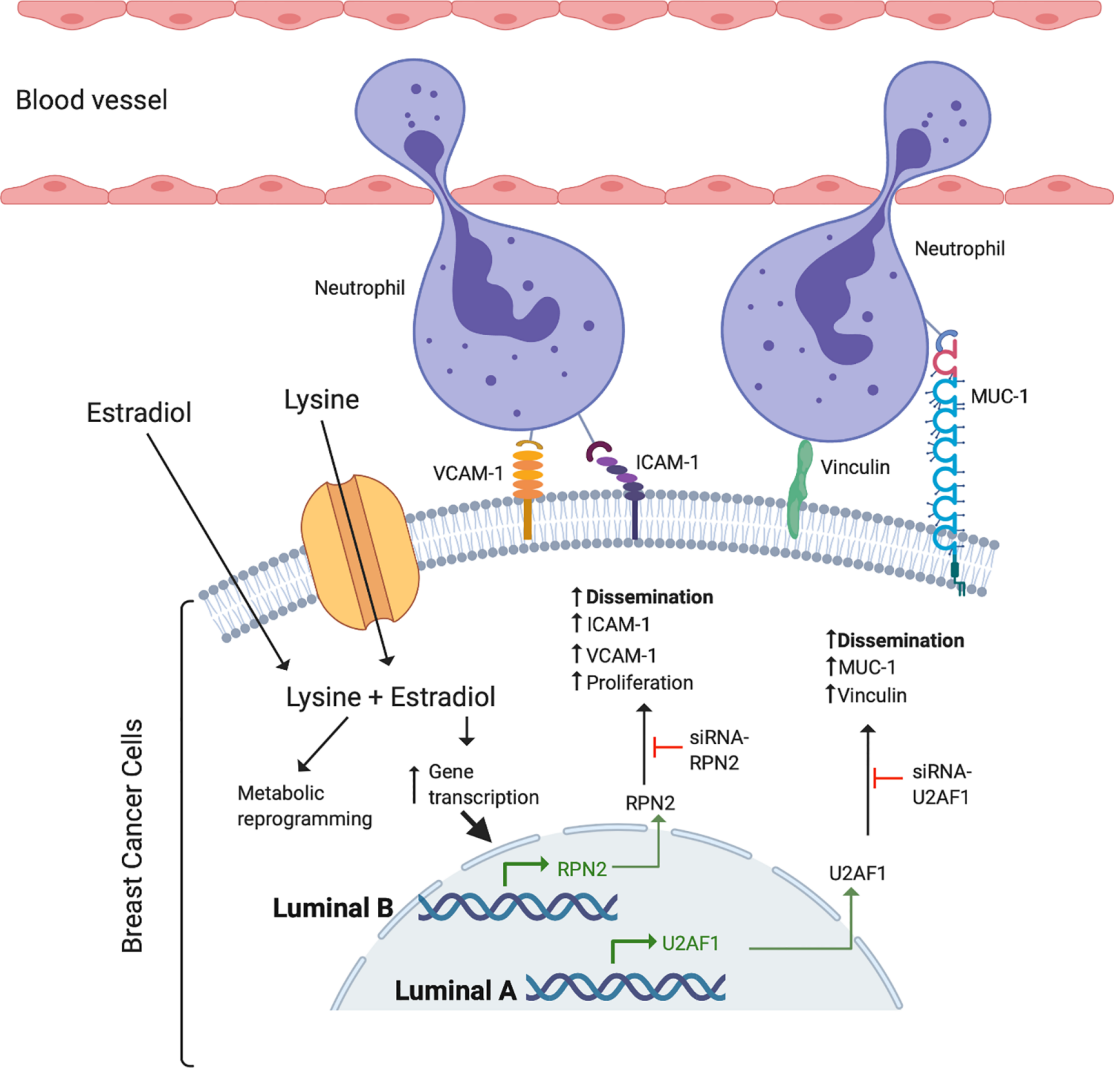
Schematic summary of the effects of lysine and estradiol on mechanisms involved in the regulation of ER+ cancer cells and neutrophil interactions resulting in increased ability of cancer cell dissemination.

Despite the key role of EAAs in the pathophysiology of many diseases including BC, the consumption preference of BCs *in vivo* and their role in the metastatic process of BC has been poorly studied. Here we show that, compared to normal adjacent breast tissue, human ER+ BC exhibited significantly decreased levels of five EAAs with lysine being the most utilized *in vivo*. Lysine, in presence of E2, significantly increased neutrophil-induced dissemination of ER+ BC cells *in vivo* in zebrafish. Proteomic data revealed that lysine, in presence of E2, induced metabolic reprogramming including upregulation of U2AF1 and RPN2 in 3D cultured ER+ BC mammospheres. Knockdown of U2AF1 decreased migration in MCF-7 cells and knockdown of RPN2 decreased proliferation in T47D cells *in vitro*, and both proteins affected neutrophil-mediated dissemination *in vivo* in the zebrafish model of metastasis *via* effects of integrin expression and focal adhesion area as summarized in **Figure 7**. In clinical data-sets, these proteins decrease overall survival of ER+ luminal A and luminal B BC patients, respectively, suggesting clinical relevance of our findings.

Lysine is one of the most abundant EAA in human blood and one of the most important proteinogenic EAAs (32, 33). We show here that lysine also was the most abundant EAA extracellularly in human breast tissue corroborating previous results in human blood. In cancer, lysine modifications and lysine metabolites have been reported to be key drivers in the carcinogenic process and to enhance tumor aggressiveness *in vitro* (34, 35). Additionally, lysine has been shown to be important in the progression of murine BC as a low lysine diet retarded tumor growth at early stages (36). Our present results support these deleterious effects of lysine as the aggressiveness of both luminal A and luminal B ER+ BC increased after lysine exposure. However, lysine exposure *per se* exhibited limited effects but in presence of estradiol the dissemination capacity increased significantly compared to either treatment alone. Our data also support previous findings that metabolic reprogramming may depend on several intrinsic cues such as breast cancer subtype, ERα/β expression and p53 mutation state as lysine exposure affected different mechanism leading to increased dissemination in luminal A and luminal B BC cells respectively (37).

The metabolic reprogramming in cancer cells might also have implications for other cell types in the tumor microenvironment including infiltrated immune cells. A nutrient depleted microenvironment may facilitate infiltration and phenotypically changes of innate immune cells in cancers (37). Once in the cancer tissue, the innate immune cells are key in increasing the metastatic capacity of the cancer cells (38). Attachment of otherwise low metastatic BC cells to innate immune cells *via* integrins such as ICAM-1, VCAM-1, MUC-1, and focal adhesion enhance their dissemination ability and thereby the metastatic capability (15, 39–41). Our present data support these findings as lysine exposure, in presence of E2, upregulated the expression of several proteins, which in turn affected the expression of integrins and focal adhesion leading to altered dissemination of the cells. Lysine exposure affected luminal A and luminal B BC cells differently. In luminal A BC lysine in presence of estradiol upregulated the expression of U2AF1 protein. U2AF1 has been reported to be one of the most common spliceosome factors affected by somatic point mutations that induce alternative mRNA splicing and contribute to breast cancer development and metastases (42, 43). Knockdown of U2AF1 decreased cell migration *in vitro* supporting previous studies of this protein in cancer progression (42). Additionally, knockdown of U2AF1 also decreased the neutrophil dependent cancer cell dissemination *in vivo*, which has not been previously reported, supporting its role in the metastatic process. Increased expression of U2AF1 was associated with decreased survival of luminal A BC in clinical databases further supporting a clinical relevance of our data.

In luminal B BC cells, exposure to E2 and lysine resulted in increased expression of RPN2, which also correlate with poor overall survival of ER+ luminal B BC patients. In line with our results, RPN2 has been shown to be highly expressed in BC stem cells, promoting BC initiation and metastasis by stabilization of mutant p53, and participating in EMT transition and drug resistance (44, 45). Knockdown of RPN2 counteracted neutrophil-mediated dissemination *in vivo* by decreasing the expression of ICAM-1 and VCAM-1 integrins. Surprisingly, knockdown of RPN2 resulted in increased expression of MUC-1 and it would be expected that this in turn would have led to increased dissemination. However, our data suggest that this integrin does not play a role in the dissemination of the cells.

To be able to determine how an individual EAA affected the proteome of the BC cells, we first cultured the mammospheres in EAA deprived culture medium with added lysine. Under these conditions we determined that lysine in presence of E2 induced metabolic reprogramming including upregulation of U2AF1 and RPN2. To evaluate whether these proteins were relevant in a more physiological environment, complete nutrient cell culture media was used in the subsequent experiments. Indeed, we found that also during complete nutrient conditions U2AF1 and RPN2 affected proliferation, migration *in vitro* and neutrophil-mediated dissemination *in vivo* in the zebrafish *via* effect of integrin expression and focal adhesion area.

Metastatic BC is an incurable disease. The risk of metastatic disease is higher for ER- BC in the short-term perspective. However, 10 to 15 years after initial diagnosis the risk of relapse and death are higher in ER+ BC (46). It has previously been assumed that ER+ BC will change its phenotype into ER- during metastases but recent data clearly show that in human BC, the majority of ER+ primary BC maintains ER expression at the metastatic site (47). Consequently, models that allow for studies of ER+ BC dissemination are key. In all murine model of spontaneously metastatic BC, ER+ primary tumors will become ER- at the metastatic site. Because of this we used the zebrafish for the metastatic studies as this model allows for investigations of mechanisms involved in ER+ BC metastasis (27, 28, 48).

We conclude that lysine is the most utilized EAA in human ER+ BC *in vivo*. Lysine, in presence of E2, induced the expression of U2AF1 and RPN2, which contributed significantly to ER+ BC cells dissemination *via* interactions with neutrophils. According to the KMP online BC database, U2AF1 and RPN2 proteins correlate with poor overall survival in ER+ luminal A and luminal B BC patients, respectively, but not in ER- BC patients, confirming their clinical relevance and subtype specificity. Restricting dietary intake of lysine, blocking its uptake in cancer cells or targeting overexpressed proteins by lysine may be a potential therapeutic approach in decreasing the risk of metastases of ER+ BC.

## Data Availability Statement

The mass spectrometry proteomics data have been deposited to the ProteomeXchange Consortium *via* the PRIDE (49) partner repository with the dataset identifier PXD022008.

## Ethics Statement

The studies involving human participants were reviewed and approved by The regional ethical review board of Linköping. The patients/participants provided their written informed consent to participate in this study. The animal study was reviewed and approved by Animal ethics committee at Linköping University.

## Author Contributions

GR performed experiments, wrote manuscript and analyzed data. AA performed experiments and wrote manuscript. MT analyzed data and wrote manuscript and CD performed the microdialysis investigations, analyzed data, conceived, designed, supervised the study and wrote manuscript. All authors contributed to the article and approved the submitted version.

## Funding

This work was supported by grants to CD from the Swedish Cancer Society (2018/464), the Swedish Research Council (2018–02584), LiU-Cancer, and ALF of Linköping University Hospital.

## Conflict of Interest

The authors declare that the research was conducted in the absence of any commercial or financial relationships that could be construed as a potential conflict of interest.
