# Supplementary material for: Lysine in Combination With Estradiol Promote Dissemination of Estrogen Receptor Positive Breast Cancer via Upregulation of U2AF1 and RPN2 Proteins
Source: Front Oncol. 2020 Nov 30;10:598684. doi: 10.3389/fonc.2020.598684 (PMC7734348; doi:10.3389/fonc.2020.598684)
Supplement: Supplementary file 3 [file DataSheet_3.docx]

Supplementary Material

# Supplementary Figures

**Supplementary Figure 1. Western blot analysis of siRNA transfected MCF-7 and T47D cells.** Equal amount of siRNA transfected MCF-7 and T47D cell lysates were loaded onto polyacrylamide gels, transferred to PVDF membranes, and incubated with specific antibodies as described in materials and methods. **(A),** Analysis of U2AF1 and RPN2 expression in lysates of siRNA-transfected MCF-7 and T47D cells to confirm knockdown of proteins. **(B),** Analysis of ICAM-1, VCAM-1 and MUC-1 expression in negative control siRNA (siRNA-C) and siRNA-U2AF1 transfected MCF-7. **(C),** Analysis of ICAM-1, VCAM-1 and MUC-1 expression in negative control siRNA and siRNA-RPN2 transfected T47D. Membranes were stripped to analyze GAPDH loading control. Membranes with their respective GAPDH loading controls are separated in black rectangles. Western blot bands shown in article are highlighted in red rectangles.

**Supplementary Figure 2. Proteomic and Reactome analysis of MCF-7 and T47D mammospheres treated ± lysine ± estradiol.** Mammospheres with ER+ MCF-7 and T47D cells were cultured ± lysine (Lys) ± estradiol (E2) for 5 or 3 days, respectively, and proteome analyzed using LC-MS as described in materials and methods. **(A),** Volcano plot of identified proteins in Lys vs Control treated MCF-7 mammospheres (n=6). Proteins with infinite (INF) fold change (FC) are shown in a separate table. Two-tailed Student’s t-test (*P <* 0.05). **(B),** Reactome analysis of significantly down- and upregulated proteins (*P <* 0.05) by Lys treatment compared to control in MCF-7 mammospheres. **(C),** Volcano plot of identified proteins in E2 vs Control treated MCF-7 mammospheres (n=6). Downregulated proteins with zero-fold change are shown in a separate table. Two-tailed Student’s t-test (*P <* 0.05). **(D),** Reactome analysis of significantly down- and upregulated proteins (*P <* 0.05) by E2 treatment compared to control in MCF-7 mammospheres. **(E),** Volcano plot of identified proteins in Lys vs Control treated T47D mammospheres (n=6). Proteins with zero and INF fold change are shown in a separate table. Two-tailed Student’s t-test (*P <* 0.05). **(F),** Reactome analysis of significantly down- and upregulated proteins (*P <* 0.05) by Lys treatment compared to control in T47D mammospheres. **(G),** Volcano plot of identified proteins in E2 vs Control treated T47D mammospheres (n=6). Proteins with zero and INF fold change are shown in a separate table. Two-tailed Student’s t-test (*P <* 0.05). **(H),** Reactome analysis of significantly down- and upregulated proteins (*P <* 0.05) by E2 treatment compared to control in T47D mammospheres. Volcano plots show code numbers corresponding to differentially affected proteins, protein names with their respective code number are shown in Supplementary data 1 and 2.

**Supplementary Figure 3. Neutralization of MUC-1 did not affect T47D cells dissemination *in vivo*.** Dil-labeled T47D cells were injected in presence of estradiol (E2) 1nM into transgenic zebrafish embryos with green fluorescent blood vessels in presence of an anti-MUC-1 antibody (αMUC-1) and its respective isotype control (Iso Ctrl) and analyzed as described in supplementary methods. Scale bar = 100 μm. Data is presented as Mean ± SEM. BV = blood vessels. Arrows show disseminated T47D cells.
